# Complete Chloroplast Genome Sequence of a Black Spruce (Picea mariana) from Eastern Canada

**DOI:** 10.1128/MRA.00877-20

**Published:** 2020-09-24

**Authors:** Theodora Lo, Lauren Coombe, Diana Lin, René L. Warren, Heather Kirk, Pawan Pandoh, Yongjun Zhao, Richard A. Moore, Andrew J. Mungall, Carol Ritland, Jean Bousquet, Steven J. M. Jones, Joerg Bohlmann, Ashley Thomson, Inanc Birol

**Affiliations:** a Canada’s Michael Smith Genome Sciences Centre, BC Cancer, Vancouver, BC, Canada; b Department of Forest and Conservation Sciences, University of British Columbia, Vancouver, BC, Canada; c Canada Research Chair in Forest Genomics, Université Laval, Quebec City, QC, Canada; d Faculty of Natural Resources Management, Lakehead University, Thunder Bay, ON, Canada; e Michael Smith Laboratories, University of British Columbia, Vancouver, BC, Canada; Vanderbilt University

## Abstract

Here, we present the chloroplast genome sequence of black spruce (Picea mariana), a conifer widely distributed throughout North American boreal forests. This complete and annotated chloroplast sequence is 123,961 bp long and will contribute to future studies on the genetic basis of evolutionary change in spruce and adaptation in conifers.

## ANNOUNCEMENT

Global climate change is predicted to impact the growth of Picea mariana (black spruce), a dominant species of significant ecological and economic importance in Canada’s boreal forests (1, 2). Black spruce has demonstrated local adaptations to climate (3). Determining the genetic and molecular bases of these adaptations can provide valuable insights into mitigating climate change effects on Canada’s forests (2, 3).

An unannotated black spruce chloroplast draft genome assembly with several gaps was submitted to GenBank (accession number LT727842.1) in 2018. Here, we present a complete and annotated black spruce chloroplast genome sequence from a different genotype.

A black spruce needle tissue sample (genotype 40-10-1) was collected in Thunder Bay, Ontario (50°57′39.96″N, 90°27′20.16″E; elevation 741 m). Following nucleus purification, genomic DNA was extracted by Bio S&T using a cetyltrimethylammonium bromide (CTAB)/chloroform method, yielding 60 μg of high-quality purified DNA (4, 5). A sequencing library was prepared using the Chromium linked-read platform from 10X Genomics (5) and sequenced with paired-end 150-base pair reads on an Illumina HiSeq X instrument at Canada’s Michael Smith Genome Sciences Centre.

One lane of sequencing data, consisting of 428,820,113 read pairs, was used to assemble the chloroplast genome. After trimming adapters using Trimadap vr11 (6), subsets were sampled (*n* = 0.75, 1.5, 3, 6, 12, 25, 50, and 200 million read pairs) to reduce the noise from nuclear and mitochondrial DNA.

Each subsample was assembled with ABySS v2.1.0 (7) using various *k*-mer sizes (*k *= 64 to 104, step 8) and *k-*mer count thresholds (*kc *= 3 and 4). Chloroplast sequences in the assemblies were extracted from BWA-MEM v0.7.17 (8) alignments of scaffolds to the reference white spruce chloroplast genome (genotype WS77111; GenBank accession number MK174379) (9) and evaluated with QUAST v5.02 (10). The assembly with the highest NGA50 length of 42,639 bp (where NGA50 indicates the length of the shortest aligned scaffold, with all aligned scaffolds at least NG50 making up at least 50% of the target genome) and 0 misassemblies (25 million read pair subset; *k *= 104; *kc *= 4) was further scaffolded using ntJoin v1.0.1 (11), supplying the white spruce chloroplast genome as the reference and setting reference_weights=“2”. Remaining gaps in the resulting scaffold were filled using Sealer v2.2.3 (12) with multiple values of *k* (*k *= 70 to 120, step 10), and the assembly was polished using Pilon v1.23 (13) with --diploid --fix all options. Approximately 700 bp on the two ends of our assembly were successfully recovered by supplying the 3′ and 5′ ends of our draft to Sealer v2.2.3 (12) using the abovementioned parameters, yielding a complete chloroplast genome. Finally, BLAST v2.10.0 (14) was used to adjust the start position for consistency with previously published chloroplast genomes. Note that default parameters were used unless otherwise specified.

**FIG 1:**
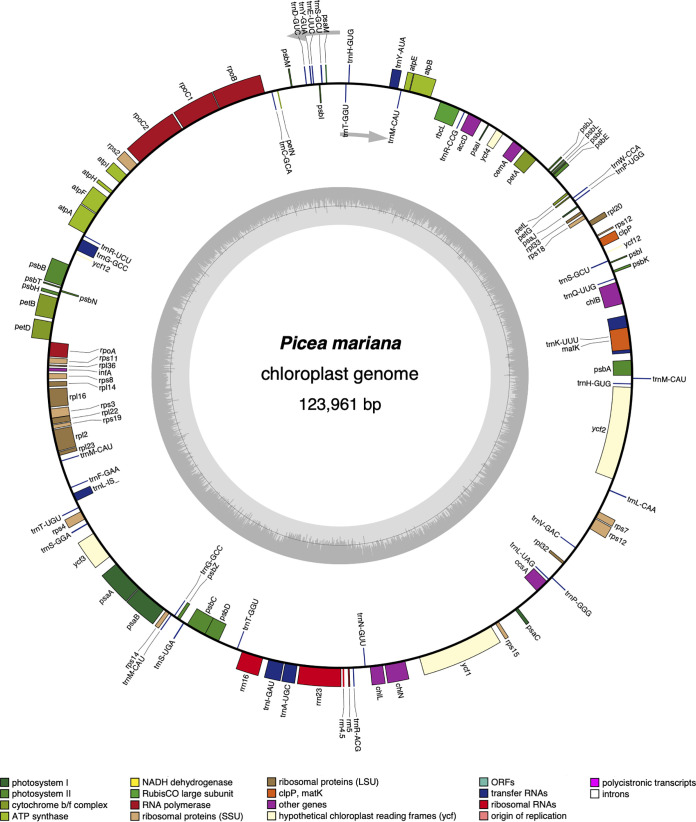
The complete chloroplast genome of *Picea mariana* genotype 40-10-1. The *Picea mariana* chloroplast genome was annotated using GeSeq and plotted using OGDRAW (16). The inner gray circle illustrates the GC content of the genome, and the outer circle shows the annotated genes as rectangular boxes with labels, colored by functional categories. The arrows indicate the direction of transcription for each DNA strand.

The complete *Picea mariana* chloroplast genome is 123,961 bp long with a GC content of 38.70%. Using GeSeq v1.79 (15), with several *Picea* sp. chloroplast genomes as references, we successfully annotated 114 genes, including 74 protein-coding, 36 tRNA-coding, and 4 rRNA-coding genes (Fig. 1). Due to a frameshift mutation, *psbZ* was annotated as a pseudogene. Also, the annotations of *petB*, *petD*, and *rpl16* were corrected manually.

Offering this chloroplast genome to the community will enrich public genomic repositories of spruce species, facilitate research on climate adaptation, and contribute to the development of forest management policies.

### Data availability.

The complete chloroplast genome sequence of *Picea mariana*, genotype 40-10-1, is available from GenBank under accession number MT261462, and the raw sequencing reads are available from the SRA under SRX7890468 and SRR11284755. The annotations used as references include those from Picea abies (NC_021456), Picea asperata (NC_032367), Picea engelmannii (NC_041067), Picea glauca genotype WS77111 (MK174379), Picea morrisonicola (NC_016069), Picea sitchensis (KU215903), Picea chihuahuana (NC_039584), Picea crassifolia (NC_032366), and Picea jezoensis (NC_029374).
